# Long-Standing Themes and Future Prospects for the Inspection and Maintenance of Façade Falling Objects from Tall Buildings

**DOI:** 10.3390/s22166070

**Published:** 2022-08-14

**Authors:** Michael Y. L. Chew, Vincent J. L. Gan

**Affiliations:** Department of the Built Environment, National University of Singapore, Singapore 117566, Singapore

**Keywords:** automated inspection, building façade, laser scanning, computer vision, deep learning, 3D reconstruction, information modelling, design optimisation

## Abstract

The increasing number of accidents arising from falling objects from the façade of tall buildings has attracted much attention globally. To regulators, a preventive approach based on a mandatory periodic façade inspection has been deemed as a necessary measure to maintain the functionality and integrity of the façade of tall buildings. Researchers worldwide have been working towards a predictive approach to allow for the assessment of the likely failure during some future period, by measuring the condition of the façade to detect latent defects and anomalies. The methods proposed include laser scanning, image-based sensing and infrared thermography to support the automatic façade visual inspection. This paper aims to review and analyse the state-of-the-art literature on the automated inspection of building façades, with emphasis on the detection and maintenance management of latent defects and anomalies for falling objects from tall buildings. A step-by-step holistic method is leveraged to retrieve the available literature from databases, followed by the analyses of relevant articles in different long-standing research themes. The types and characteristics of façade falling objects, legislations, practices and the effectiveness of various inspection techniques are discussed. Various diagnostic, inspection and analytical methods which support façade inspection and maintenance are analysed with discussion on the potential future research in this field.

## 1. Introduction

**Figure 1:**
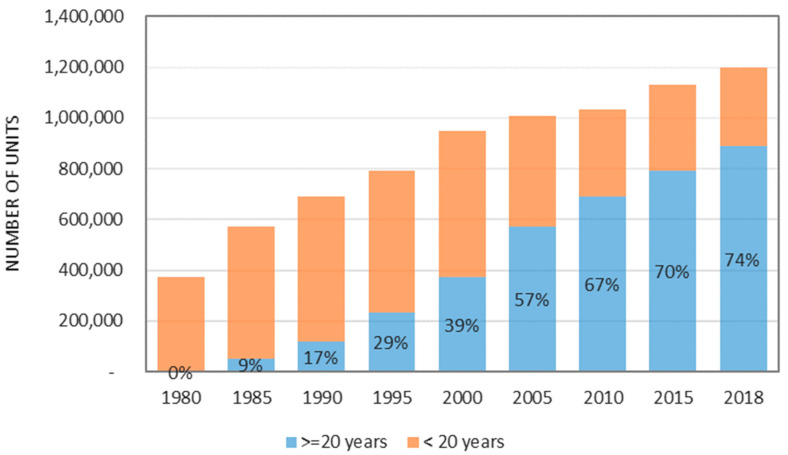
Percentage of public residential buildings in Singapore exceeding the age of 20 years [2].

A regular building inspection and condition assessment have been deemed as a necessary measure to maintain the functionality and integrity of buildings and civil infrastructures. Since there is an increasing number of old buildings, the exposure of façades persistently experiencing adverse outdoor environmental conditions catalyses the degradation [1]. The percentage of public residential buildings in Singapore exceeding the age of 20 years was 74% [2] (see Figure 1). The city has reported more than 90 incidents in the past three years where parts of the façades fell off. It is expected that more and more façade defects and incidents of falling objects from heights will incur, leading to serious public safety issues [2]. As such, structural health monitoring is becoming an indispensable inspection task during façade condition assessment as falling objects from tall buildings can cause potential damage to the public and trigger structural safety considerations [3]. Periodic monitoring and building inspections are necessary to rationally secure the safety of building components [4]. This leads to the necessity of new knowledge about the types and characteristics of façade falling objects, the critical factors affecting the falling and the effectiveness of various inspection techniques.

The current practice relies on visual inspection by certified inspectors. The surface defects detected during each inspection are documented by photos and sketches. As such, the conventional inspection practices are insufficient to holistically understand the building condition at the reviewing stage. To resolve this problem, researchers have leveraged unmanned aerial vehicles (UAVs) to support automatic visual inspection [5]. Since UAVs have relatively lower payloads, unmanned ground vehicles (UGVs) or ground robots were more easily stabilised to carry advanced sensing devices such as Light Detection and Ranging (LiDAR) laser scanners for point cloud acquisition [6]. Such 2D images or 3D point clouds were further used to identify building defects and analyse potential damages. This included the identification of concrete spalling defects using laser scanning [7], concrete surface defect quantification with UAV-based laser point clouds [8] and change detection and deformation monitoring [9]. For instance, image data obtained with UAVs were used to detect different types of concrete cracks on buildings [10]. The use of infrared thermography to capture delamination defects before crack formation was also investigated [11,12]. Recent studies have also focused on using point clouds for quantifying building defects [13]. Furthermore, image-based 3D reconstruction was explored to support building condition evaluation and damage assessment [14].

However, previous relevant studies focused on the development of defect/feature detection algorithms for identifying surface defects. A systematic evaluation of the current practices on façade inspection and maintenance is still missing. Furthermore, there will be public safety issues because more incidents of falling objects from heights may incur as a result of old buildings reaching their serviceability life. By far, there is still a lack of systematic reviews to discover the long-standing research themes and prospects for inspecting and maintaining façades to prevent falling objects. 

Therefore, this paper aims to review the state-of-the-art literature on the automated inspection of building façades, with a focus on the detection, assessment and maintenance of falling objects, which can trigger public safety issues. A holistic method is leveraged to retrieve the available literature from databases, followed by analyses of relevant articles on different long-standing themes. The common elements/components with a high risk of falling, as well as the type of detection methods, are discussed. The information in the available articles is also analysed to reveal future research needs towards falling object inspection and maintenance. Various diagnostic, inspection and analytical techniques that improve façade maintainability are summarised. The findings of this paper provide insights for determining future research in related fields. Section 2 explains the methodology for the literature search. Section 3 presents a critical discussion on the state-of-the-art methods used in the context of façade inspection. The research gaps and potential research directions are discussed in Section 4. Section 5 summarises and concludes the paper. 

## 2. Methodology

This paper presents a comprehensive review of the state-of-the-art research on façade inspection and maintenance from 2010 to 2022, giving 12 years to view the development trends over one decade. The time is from the year 2010 because there are very few relevant papers published before 2010 [15,16]. The literature retrieval was based on defect detection and inspection studies published in major literature databases such as Web of Sciences, Scopus, etc. The keywords utilised to search the published articles included specific topics in this field such as façade inspection, defect detection, surface defect, damage, building, civil infrastructure and engineering structure, which are related to the most previous relevant studies in this domain. The keyword search was deemed relevant to the interesting topics and was sufficient to cover the previous relevant studies.

A sample collection was then performed through the segregation of research articles. A portfolio of more than 50 original research articles and conference proceedings was acquired. The articles were then classified and analysed to ensure that they met the inclusion criteria, such as having a significant development in detection methods, etc. The screening process was conducted to refine the results to the relevant scope, excluding other irrelevant subject fields. During the content analysis, the outcome returned 43 significant research articles from more than 10 journals. The selected research articles were then analysed thoroughly and classified into specific categories, ensuring that their scopes were relevant to the objective of this study.

**Figure 2:**
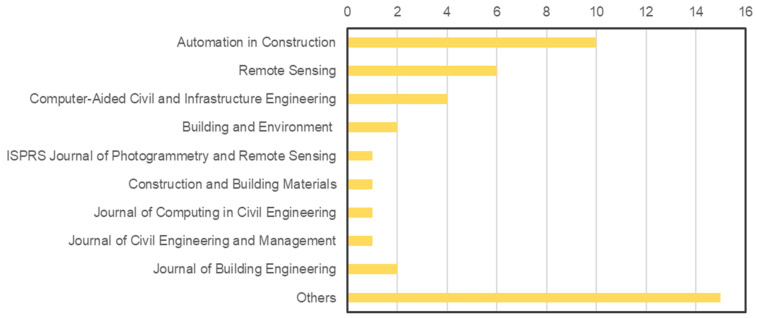
The number of articles that appeared in major journals.

**Figure 3:**
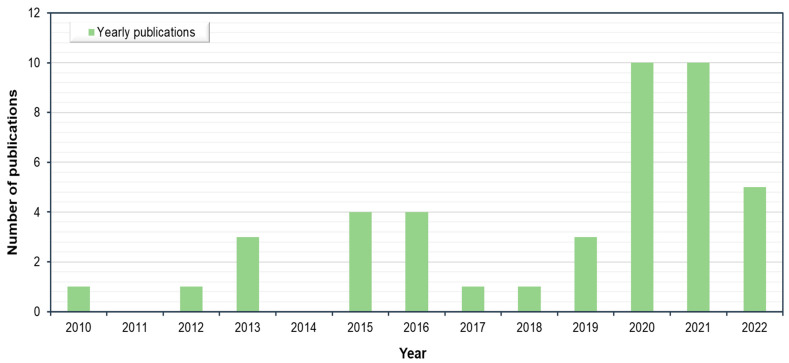
The number of articles for each year.

Figure 2 shows the overall picture of the articles, whereas Figure 3 presents the number of articles among the different research themes. The journals *Automation in Construction, Remote Sensing, Computer-Aided Civil and Infrastructure Engineering, Building and Environment, ISPRS Journal of Photogrammetry and Remote Sensing, Journal of Computing in Civil Engineering*, etc. have been found to publish more papers in this field. The amount of publications had a considerable increase over the past few decades. It can be argued that there is a growing interest in façade inspection and maintenance as the number of publications continues to increase. The literature retrieved was further analysed regarding the methods, techniques and algorithms for façade defect detection and diagnosis. Details of the findings are presented in the following sub-sections. 

## 3. Façade Defects and Inspection Practices

This section presents state-of-the-art practices for façade inspection and maintenance, including falling objects from tall buildings. Previous relevant studies on automated façade inspection are discussed to identify long-standing research themes in this field. 

### 3.1. Types of Façade Defects and Anomalies 

The serviceability of the building façade is affected by the physical property of the building materials as well as the exposed environment. Table 1 summarises the common defects and anomalies from different types of façades which potentially cause falling objects from tall buildings. The typical problems highlighted include cracking, water penetration, misalignment, discolouration, efflorescence, corrosion, etc. Concrete is one of the most common construction materials for building façades, in which case cracking, spalling, biological growth, drying shrinkage and delamination are typical surface defects that cause falling objects. The localisation and quantification of concrete cracking and spalling defects have been studied with various sensing techniques [7,10]. Other types of façade materials include brick masonry, plaster and tiling, which would lead to falling objects. In particular, their defects such as cracking, rising dampness, biological growth, efflorescence and delamination are common in tropical climates with high temperate and humidity [17]. However, a study on the design and maintenance at the outset during the planning stage for façade components is still lacking in the literature. One other potentially high fatal falling object is cladding. This involves stone cladding, metal cladding and glass cladding. The main reason for falling includes damage and cracking on the façade materials, joint or connection failures and the inadequate design and maintenance of the support system. Investigations showed that casement windows constitute 80% of the fallen windows because of the corrosion of aluminium rivets, as well as improper design, installation and maintenance [2]. As such, there is a research need to improve the identification and classification of common façade defects and anomalies.

### 3.2. Overview of Façade Inspection Practices and Regulations 

Table 2 shows the relevant global standards and legislations worldwide for façade inspection. Chicago’s (US) Department of Buildings [18] requires frequent inspections between 4 and 12 years for high-rise exterior walls and enclosures for buildings 80 feet tall and higher. The consideration of building service life relating to maintainability is incorporated into the inspection standards and protocols. For example, Cincinnati’s (US) General Inspection Programs [19] require an inspection schedule of 8 or 12 years for buildings with five stories and that are 15 years old. Likewise, buildings of five or more stories must be inspected every 5 years in compliance with Quebec’s (Canada) Safety Code from the Building Act [20]. In general, buildings with more than five stories or that are more than 75–80 feet tall require a regular inspection schedule of 4–12 years. Such inspection applies to buildings varying from 15 to 30 years old.

Façade inspection consists of two stages. The first stage is to assess the general condition of the building under inspection. Visual aids such as binocular cameras and infrared thermography cameras mounted on a drone [10,21,22] are some of the methods used for inspection. Specifically, it involves a visual inspection of the entire façade area to detect anomalies for the entire building from the ground level. To streamline the management of UAV-collected information, the aerial images are integrated with a geographic information system (GIS) [23] or building information modelling (BIM) [24] to support the automated detection of the dilapidation of façade elements. The retrieval and analysis of the images are performed for detecting and documenting façade anomalies. Airborne images are processed with different image processing and detection algorithms, from which the surface detections of buildings (such as concrete cracks) are extracted and identified [10]. 

Following the visual inspection, the second stage emphasises the hands-on inspection of each elevation. In practice, at least a 10% inspection shall be conducted for each building face [25]. This requires the application of non-destructive and destructive tests to examine the severity of the defects and anomalies [26]. The inspection may include different kinds of measures (such as tapping, the partial removal of façade elements and material testing). Recommendations of remedial and maintenance measures shall then be provided based on the evaluation of façade elements.

**Table 2:** Legislations worldwide for façade inspection.

Region/Country	Standard	Description	References
ASTM, US	Standard Practice for Periodic Inspection of Building Façades for Unsafe Condition	-	[27,28]
Chicago, US	Maintenance of High-Rise Exterior Walls and Enclosures	Buildings of 80 feet tall. Inspection frequency between 4 and 12 years.	[18]
Cleveland, US	Exterior Wall and Appurtenances Inspections	Buildings with five stories or that are 75 feet tall and 30 years old. Exterior inspection every 5 years.	[29]
Cincinnati, US	Chapter 1127—General Inspection Programs	Buildings with at least five stories or sixty feet and that are 15 years old or greater. Inspection schedule of 5, 8 or 12 years for different categories of buildings.	[19]
New York, US	Local Law 11 of 1998	Buildings of six stories or more.	[30]
San Francisco, US	Building Code—Building Façade Inspection and Maintenance and Establishing Fee	Buildings of five or more stories.	[31]
Quebec, Canada	Safety Code—Building Act	Buildings of five or more stories. Inspection every 5 years.	[20]
Hong Kong	Mandatory Building Inspection Scheme and Mandatory Window Inspection Scheme	Buildings of 30 years old. Inspection every 10 years.	[32]
Singapore	Building Control Act 1989	Buildings taller than 13 m and that are 20 years old. Inspection every 7 years.	[33]

## 4. Long-Standing Research Themes 

The selected articles from the literature were further divided into common research themes. The theme categorisation followed the criteria that were set as the main objective(s) of performing a façade inspection, including the diagnosis of different building surface defects. Such filtering was deemed to provide an adequate amount of literature for analysing the current trend and prospects in this domain. Table 3 presents a summary of the literature, year of publication and description of work, as well as the automation and sensing devices used in the respective analysis. Some articles are further discussed in the following sub-sections.

### 4.1. Sensing Techniques for Façade Defect Detection

Earlier studies in this field applied different methods such as terrestrial laser scanning [36,38], imaged-based sensing [34] and thermography inspection [43] for detecting façade defects. The main differences between laser scanning and imaged-based sensing lie in the kind of data collected. Terrestrial laser scanning relies on detecting wavelengths of light radiation, whereas imaged-based sensing uses RGB images rather than collecting light wavelength data. Laser scanning allows for the coverage of a very large area, but it is computationally expensive for measurement and analysis. Imaged-based sensing is more advantageous in terms of cost and provides an accurate measurement of coordinates in spaces, but it provides limited support for measuring texture-less or weak-texture objects.

A previous study [38] leveraged terrestrial laser scanners for assessing pathologies in Villamayor stone façades. The raw point cloud data were processed and segmented to remove noise points that were not part of the Villamayor stone. Following this, an unsupervised classification was performed to recognise stone varieties and biological colonisation [38]. Regarding thermographic inspection, Edis et al. [41] presented the detection of moisture variation in façades with infrared thermography. A principal component analysis was used to analyse the time-dependent thermography data [41]. Another study [44] quantitively compared the efficiency of two thermographic inspections, namely pass-by thermography and walk-through thermography for identifying building defects. Besides laser scanning and image-based methods, vision-based recognition is another popular area of interest for new journal articles. With recent developments in experimental applications, certain amounts of façade data have been published and available for image processing to underpin the detection, segmentation and classification of façade defects or features [61,62]. Kouzehgar et al. [48] presented a convolutional neural-network-based approach for crack identification in glass crack detection. The proposed method hit an accuracy of 90% in recognising cracked glass [48]. Some research [62] on the efficiency of image recognition has explored and implemented a mask region-based convolutional neural network model to realise the automatic detection and segmentation of façade defects. The proposed method resulted in accuracy improvement for both detection and segmentation [62]. Some studies have attempted a wide area monitoring of façades using oblique aerial images [46]. For example, Duarte et al. [51] proposed the detection of seismic façade damages with aerial photography that is collected at a specific angle to the ground. It can be applied as an initial survey method covering wide geographical extents and identifying severe façade damages. Yang et al. [39] presented a method to recognise façades from large-scale urban Manhattan scenes with oblique aerial images. However, this method provided limited supports to detect small damages, such as smaller cracks or smaller areas of spalling, because of the low-resolution images used.

Although a variety of instrumentation from tapping to non-destructive sensing techniques was utilised to examine the extent and severity of the anomalies, the recognition of falling objects is still relatively new and largely unexplored in the literature. The scientific challenge underlying this issue is the classification and diagnosis of the severity of various façade elements that might result in falling. This requires a deeper understanding of the design, construction, environment and structural/architectural materials to identify and evaluate the extent of the damage on façade elements that potentially cause falling from heights.

### 4.2. Automated Methods for Façade Inspection and Maintenance

For a building with widespread defects observed, a full façade investigation of localised areas or the whole building might be needed. This involves a visual inspection of the entire façade area to assess the condition of the entire building façade elements from the ground level. This process can be time demanding and labour intensive, and therefore the use of UAVs has attracted attention to automate façade inspection [10,23]. This is evident in Table 3, where very few studies have applied drones/robots to assist the inspection before 2020 whereas UAVs were leveraged commonly since 2020 to support visual inspections. In this sense, Roca et al. [22] were one of the early attempts to leverage UAVs and low-cost scanning sensors to automatically obtain geometric data including unreachable areas in buildings. The 3D point cloud data of a building façade element were generated automatically from the visual and depth images collected by the outdoor inspection aerial unit [22]. However, motion blur can arise due to the vibrations of UAVs during the flight. As such, a deep-learning-based deblurring model was studied to resolve motion blur due to the excessive vibrations of UAVs amid crack detection [3]. Besides UAVs, some researchers [48] have implemented façade-cleaning robots equipped with deep-learning-based detection algorithms for crack identification. The image data collected were more effective and accurate in detecting cracks on the glass façade while reducing human engagement in the time-consuming process. 

Robotic and automation technology has undoubtedly reduced the time and manpower required to complete the inspection; this process, however, generates increasing amounts of image data. The storage and management of large amounts of collected façade images is another matter of concern [23]. In this regard, GISs provide support for the documentation of façade defects and anomalies. Chen et al. [23] presented the geo-registering and managing of UAV-collected images to the 2D GIS spatial model for façade inspection. The geo-registrations of UAV images enable the more accurate mapping of recognised and existing data collection for building façades while documenting façade features in a spatiotemporal-based documentation platform for life cycle maintenance. A GIS-based data management platform facilitates the retrieval and analysis of building façade data. This involves laser point clouds, high-definition images and infrared data for documenting façade anomalies [50].

BIM is another paradigm of digital management that has been actively explored by researchers in recent years for the visual inspection of buildings and infrastructures. Being an integrated digital model that collects geometric, semantic and topological data throughout the life cycle of a project [64], there have been developments for BIM usage in building inspection. Truong-Hong et al. [35] developed a framework and algorithms for detecting building boundary features and converting point cloud data into a solid model. However, a solid model did not contain the necessary semantic information (e.g., materials) for buildings. In this sense, Mill et al. [36] presented 3D terrestrial laser scanning and total station surveying for creating a BIM, on top of the façade defect detection. The BIM model contained not only accurate information related to geometries but also inner spatial relationships and semantics for materials that can be leveraged for the condition assessment regarding façade maintainability. In the meantime, research with a particular focus on BIM has also received greater scholarly attention in recent years, specifically for the processing means of BIM-based information storage and enrichment. Additionally, the information in a BIM model has been extracted for the automatic UAV inspection of building surfaces [57]. The imagery data and laser point clouds were not only used to detect building surface defects but also to generate 3D models for better digital management of the building information for maintenance planning. 

### 4.3. Façade Defect Assessment and Diagnosis

An emerging field in deep learning is image segmentation and detection work, as researchers are starting to utilise a wide variety of new algorithms that have been explored in computer vision. Some have explored using deep learning as an approach to provide more accurate façade defect segmentation [26,53]. Specifically, Guo et al. [26] proposed a rule-based deep learning method to provide evaluations containing the type, location, quantity and size of the façade defects. Many studies have cited the limited data for processing and further segmentation. As such, the same authors [53] proposed a semi-supervised learning algorithm that improved the classification accuracy from 79.26% to 84.36% with a smaller amount of labelled data. Alternatively, another study [60] proposed a bounding-box object augmentation method for object detection in residential building façades. A faster region-based convolutional neural network model was tested on the augmented training dataset and exhibited better performance for feature detection than that using the original dataset [60]. 

While numerous studies were devoted to the interest of deep learning, some papers have investigated the feature detection of façade elements [24,45]. For example, Zolanvari et al. [45] studied the slicing method applied to the identification of curved façades and window boundaries/features and converted point clouds into a solid model. Yin et al. [24] used an automatic layer classification method for floor plan and elevation detection to enable the reconstruction of a 3D (façade) model. Some studies also explored the effectiveness of different sensing techniques to identify the features of façade elements [49,63]. Masieroa et al. [49] presented a support vector machine classifier to process terrestrial laser scan data for detecting small damages on brick façades. Another study proposed active infrared thermography for the segmentation of defect areas and to achieve automation in thermal image processing [63]. These alternative models and techniques are useful to detect visual features on façade elements, creating domain recognition and learning models to develop classification algorithms for falling objects in the future. In general, most papers using multiple algorithms are interested in comparison to determine the best precision detection and segmentation. However, as most algorithms are stuck in a limited application mainly to find better accuracy, more exploration into damage and condition assessment can be a new area of interest. Most of the studies related to this topic are still in infancy and more research efforts are required to explore the diagnosis of the severity of façade defects that might result in falling, taking into consideration the design, construction, material property and environmental impacts.

Lessons learnt from past and present studies indicated an increasing trend in the sensing techniques, automation methods and algorithms for the effective detection and diagnosis of façade defects. Recommendations in the future include the consideration of new methods and algorithms to inspect different kinds of falling objects at the outset of the building maintenance stage, as well as the assessment of the severity of the damage to façade elements which potentially cause falling from heights. A recommended benchmark for the risk index was proposed [2,25] to classify the potential damage of different falling objects, based on their severity. Risk indexes are classified as high, medium and low with different levels of toleration. Condition and damage assessment for the falling object is still much needed to identify and document the risk index for future maintenance and inspection to ensure the risk is kept to a minimum. 

## 5. Future Prospects

In this section, we further analyse and discuss the findings and their implications in a broader context. Future research directions in this field are highlighted as follows.

### 5.1. Fully Automatic Façade Inspection

There have been several studies utilising sensing technologies, with the aid of ground robots, UAVs or UGVs to inspect the dimension and surface defects of buildings and civil infrastructures. Three main types of sensors were commonly used in previous relevant studies, which were 3D laser scanners, binocular sensors and 2D cameras [65]. A past review [9] showed that terrestrial laser scanning has been leveraged to assist deformation analysis in structures, and more efforts are needed for point cloud processing for detecting change, the incorporation of deformation measurements, etc. With advances in digital imaging and image processing, there is an increasing volume of research articles on image-based methods for building and construction, such as object detection and recognition [66]. Despite laser scanning or image-based sensing, a combinational method of texture-based reasoning and colour-based reasoning were proposed in previous studies [67]. Infrared thermography is gaining attention as an important building inspection and diagnostic tool.

With the advancement of robotic technology, automation-enabled inspection and health monitoring for buildings and infrastructures have attracted more and more attention. Yu et al. [68] integrated a mobile robot system with a crack detection method to automate concrete crack inspections in tunnels. Similarly, Menendez et al. [69] presented an autonomous robotic system for tunnel structural inspection and assessment. The authors designed and developed a multi-degree-of-freedom robotic system, consisting of a mobile vehicle, a high-precision robotic arm and an ultrasonic sensor to measure the width and depth of detected cracks [69]. Such robotic technology usually comes with one of the sensing devices for automatic inspection. In this sense, researchers have leveraged computer-vision-based image-processing techniques for detecting defects and conducting condition assessments [70]. The robotics and automation-enabled methods, facilitated by a path planning algorithm, are promising because the conventional manual inspection was very time demanding and often not safe [71]. 

Automated defect detection for building façades and civil infrastructures are practically relevant research directions. Building façade inspection usually leverages drones rather than ground robots and UGVs. The reason is that tall buildings are vertical structures where a UAV inspection can reach many inaccessible areas without risk for the operator [72]. At the current stage, UAV control for façade inspection relies much on human operators. This is because the façade images need to be taken from different perspectives, occlusions and illuminations [61]. As compared to infrastructures, the heterogeneous textures, non-building elements (e.g., doors, air-conditioning) and obstacles in urban scenes increase the difficulty of detecting the location and shape of façade elements. Civil infrastructures (e.g., bridges, roads, tunnels) are horizontal structures where inspections can leverage either UAVs or ground robots. In this regard, some researchers [73] have proposed 3D path planning using a LiDAR-equipped UAV for bridge inspection considering the potential locations of defects. Additionally, the autonomous operation of ground robotic systems has been found to reduce the risk of the inspector and save the time and manpower required to complete the inspection. Research into automation-enabled inspection especially in the aspect of buildings and civil infrastructures requires more effort. To prompt automation-enabled façade inspection, using remotely operated technologies (such as vision-based measurement [74], heterogeneous robotic system [75], genetic-algorithms (GA)-based flight path optimisation [57], etc.) for autonomous drone localisation, motion planning and control, while keeping the operators informed of the inspection, is much needed in the future. These methods need to be modified and transferred to civil infrastructure, taking into consideration their distinctive characteristics and geometric features.

Furthermore, the applicability and accuracy of state-of-the-art frameworks are another matter of concern. Earlier studies were applied mostly to elements with planar and regular geometries. For example, Truong-Hong et al. [35] introduced a flying voxel method with Delaunay triangulation for extracting façade and window boundary point cloud data for reconstructing a geometry-compatible façade. Few studies have been performed for measuring irregular surfaces or geometrics, which are sometimes adopted for façade elements. In this sense, Zolanvari et al. [45] presented detection algorithms to identify the non-rectilinear building boundaries/features and convert the point cloud data into a solid model for computational modelling. While some research has studied curved geometries, more generalised methods and algorithms are still much needed for measuring the as-is dimensions of non-planar elements. The accuracy of the quality assessment is another issue. A review article [65] indicated that previous relevant studies on quality assessment mostly fall between a 5 mm and 30 mm accuracy. The discrepancy might be larger for detecting the surface defects, such as falling objects, due to their complex features. In general, an improvement in the applicability and inspection accuracy of the current methods is still much needed for as-is measurements amid façade inspection. This will create an accurate generation of as-is 3D models for future renovations of façade elements to prevent falling objects and other defects. 

### 5.2. 3D Modelling of Façade Defects for Maintenance Management

BIM is used as a 3D modelling tool to store and retrieve required information for buildings and infrastructures for better information management, building planning and facilities maintenance. Numerous publications have reviewed important current BIM-related investigations to gain an understanding of BIM in various disciplines. Research papers, for instance, looked at the use of BIM in construction projects from diverse perspectives, such as structural analysis and design computation [76], and as-built data collection [77,78], with the aid of laser scanning or image-based methods. The as-built point cloud data were usually processed by machine learning algorithms to automatically segment and classify the building components [79], which in turn assist the generation of 3D BIM models. In this sense, Brilakis et al. [80] and Tang et al. [81] were the early attempts to study the automatic generation of as-built BIM through laser scanning data. Since the authors emphasised that BIM modelling requires not only geometric information but also considerable improvements in semantic information to create an improved data exchange across various applications, other studies have explored laser scanner data for the automatic generation of a semantically rich information model [82].

Despite the benefits of 3D modelling, the current BIM framework still needs substantial improvement for building/structural health monitoring as well as automatic façade inspection. One critical review of the BIM literature [83] indicated that previous research areas mainly cover “BIM Adoption and Standardisation”, “BIM Programming”, “Image Processing”, “Laser Scanning”, “Augmented Reality” and “Collaborative Environments and Interoperability”. BIM-enabled applications for building façade inspection and condition assessment (such as falling objects) remain in infancy and require more research efforts. In this sense, Mill et al. [36] presented the use of laser scan point clouds for creating BIM for the digital management of façade damage detection, contributing to the domain of façade defect identification. Sacks et al. [84] proposed a BIM workflow for the interoperable design and construction of architectural precast façades, contributing to the information management of façade construction. Recent studies have explored the mapping and modelling of the defect data collected from UAV images in the BIM environment [85].

While existing 3D BIM models can store geometries and very detailed semantic information about a building [86], they are not semantically rich enough to represent façade defects including information related to falling objects. The existing BIM data schemas, Industry Foundation Classes (IFC), still lack the entities and property sets required for façade anomalies. To incorporate new semantic data as well as to improve data interoperability, initiatives were put forward by extending the IFC data schema. In this sense, Sacks et al. [87] presented the semantic enrichment of building models by devising a new procedure for compiling inference rules for the complete classification of model objects and enhancing the computation of complex geometry to enable precise topological rule processing. Specifically, for the inspection domain, Motamedi et al. [88] proposed an extended IFC data schema to systematically store various types of degradation and defect information in buildings and infrastructures. A case study is presented in which a set of interrelated defects and their relationships with other elements were modelled and visualised in BIM applications [88]. A similar BIM-based framework was proposed for damage segmentation, modelling and visualisation using IFC [89]. These studies will inspire further research into IFC data extension and IFC-compliant as-built BIM generation using 3D point cloud data to represent façade defect information amid building maintenance management. 

### 5.3. Façade Defect Diagnosis and Predictive Maintenance

There are three main maintenance strategies nowadays, namely, corrective maintenance, preventive maintenance and predictive maintenance [90]. Corrective maintenance rectifies defects or faults after the faults are diagnosed. Preventive maintenance is based on a fixed schedule for repairing the defects or degraded parts of buildings [91]. Conventional facilities management relies on corrective or preventive maintenance strategies. In this sense, Fang et al. [92] presented a computer-vision-aided inspection method based on deep learning occlusion mitigation for detecting/checking falling prevention measures of steeplejacks in an aerial environment. Wu et al. [93] proposed an integrated information management method to support the proactive prevention of falling object accidents. 

Since unexpected failures in façades can cause very significant consequences and safety issues, predicting the potential façade failures and defects that may have severe impacts on public safety is needed. Klimkowska et al. [94] reviewed the methods and algorithms for image and point cloud processing for building façade 3D reconstruction. With the 3D model, predictive maintenance can be performed with AI-based condition monitoring to assess and predict the performance of a building component [95]. Zhang et al. [91] presented the integration of BIM and AI including knowledge-based reasoning and machine learning for building and infrastructure maintenance management. BIM and machine learning or other computational algorithms were leveraged to develop predictive maintenance strategies to analyse future conditions [96].

In addition, while there were numerous publications on the predictive maintenance of buildings, its applications in the field of façade maintenance (such as falling objects) were still insufficient. Vieira et al. [97] proposed a Takagi–Sugeno fuzzy model for the service life prediction of rendered façades. Another in-depth analysis of BIM connected with building condition assessment and causality analysis was provided by Alavi et al. [95]. The system architecture was proposed to automate the data transfer process between BIM and the building condition risk assessment model, supporting better decision making in façade maintainability [95]. A comparison of the life cycle costing of façade preventive and predictive maintenance scenarios was conducted [98]. These scenarios were compared by characterising their service life, minimum level of quality, maintenance operation, frequency and cost [98]. 

Very recently, the idea of BIM and machine learning was merged with digital twins for predictive maintenance in infrastructures [99]. Digital twins contain a semantic-rich model of building components that can carry out a prediction for informed decision making. However, a comprehensive description of a digital twin architecture concerns not only a virtual model but also sensing, which acquires and communicates condition data of the physical asset with the virtual system for advanced prediction and reasoning [100]. Since the concept of digital twins is relatedly new, the authors [99] highlighted that more research effort is needed to advance digital twins with machine learning techniques for building predictive maintenance management. The research effort is required to establish a unified platform wherein the sensing data acquired from façade inspection can be promptly leveraged to predict the façade service life and working condition and to generate optimal maintenance planning.

### 5.4. Data-Driven Design Optimisation for Maintainability

This section discusses the frequently considered optimisation issues in previous studies. New design-optimisation methods and algorithms have been studied to support spatial planning and structural optimisation [101,102]. Available spaces in design optimisation were represented by 3D grids with their locations and dimensions as the design variables so that the spaces were then moved freely to create new layout plans [101]. The optimal building space and topology were formulated and therefore suitable for the algorithmic search for the optimal design [102,103]. With the advancement of neural network computing, researchers have leveraged machine learning to improve design optimisation. Since machine learning makes predictions based on historical data, the procedure requires a shorter time than a conventional simulation-based evaluation of the candidate design [104,105]. Then, it is of vital importance to define the scope of the optimisation in the form of an objective function for guiding the design exploration [106,107].

When moving towards façade design, the optimisation model needs to incorporate the maintainability of the façade elements, which increases the complexity of the computational design optimisation [108,109,110]. Montali et al. [110] presented a knowledge-driven optimisation method for prefabricated façades. The optimisation process consisted of establishing a product-oriented knowledge base for designers to identify the optimal solution that considers façade architectural intents and performance requirements [110]. Another similar study [108] presented a data-driven approach for investigating façades using illuminance optimisation. Despite the data-driven design of façades, there is little consideration of maintainability in the optimisation process. Design for Maintainability (DfM) is the practice of integrating maintenance experience in the design process, taking into account the safety and economy of maintenance tasks throughout the life of a building or infrastructure [111]. A holistic methodology is needed to link/quantify the façade maintenance goals to the design optimisation process, and how it can be applied to the practical design of residential and non-residential buildings.

In addition, the DfM optimisation, taking into account the ease, safety and economic considerations throughout the building life cycle, would be most likely a complex, NP-hard problem. Therefore, inventing new solution methods for DfM optimisation is needed. Table 4 summarises the classic computational algorithms used in building design. Optimisation techniques such as linear programming [112], non-linear programming [113] and sequential linear programming [114,115] have been used in the literature to optimise different aspects of structural design. Alternative techniques including genetic algorithms [116], particle swarm optimisation [117,118], harmony search [119] and ant colony optimisation [120] were also utilised to explore optimal design solutions. The early attempts were limited to the small-scale design optimisation problem, and the computation was relatively fast because objective functions in these studies involve relatively small search spaces. 

When it comes to DfM optimisation, the choice of optimisation techniques depends on the characteristics of specific façade design problems. Hybrid algorithms provide a promising solution [121]. For instance, Masouleh [122] integrated an ant-colony-algorithm-based generator with an active learning framework to iteratively generate and evaluate design scenarios. Gan [123] proposed a BIM-based 3D geometric modelling and generative design method to automatically manipulate the geometric variations of high-rise buildings. The generative modelling technique can be further integrated with deep learning algorithms for a more advanced search for the optimal design. Generative adversarial net, which involves a generator searching for candidates whereas a discriminator evaluates the generated options, was applied for layout optimisation [124,125]. The capability of the generative adversarial net was integrated with graph-based deep learning to identify the optimal design that leads to human comfort [126]. Machine learning serves to learn from the problem structure to help control the optimum searching process. More effort is expected in the future to rigorously formulate the optimal DfM problem and invent new algorithms for DfM optimisation.

## 6. Conclusions

This paper attempts to provide a critical review of the automated inspection and maintenance of façade elements, with more emphasis on falling objects from tall buildings, which might cause public safety issues. Numerous research publications were examined from a variety of peer-reviewed journal articles. The types and characteristics of the façade falling objects, the critical factors affecting the falling and the effectiveness of various inspection techniques are discussed. The long-standing research themes for state-of-the-art scholarly articles on façade inspection were analysed in different dimensions. Specifically, previous studies focused on the research and development of three fundamental subjects, namely, sensing techniques for façade defect detection, automatic façade inspection methods and algorithms for façade detection and segmentation. The majority of past research falls into these three areas, which concerns new methods for data acquisition and feature detection. This paper then provides a complete picture of the research and reveals future needs for façade inspection and maintenance management for buildings and infrastructures. In this regard, there is growing interest in automation-enabled façade inspection, 3D modelling of façade objects, predictive maintenance and data-driven design optimisation. Various diagnostic, inspection and analytical techniques that support better façade inspection and maintainability are discussed. The findings of this paper help come up with an overall picture and provide a deeper understanding of future research.

## Figures and Tables

**Table 1:** Common defects and anomalies from different types of façades.

Type of Façade	Common Defects and Anomalies	Examples
Concrete	Crack, spalling, biological growth, drying shrinkage, concrete delamination, etc.	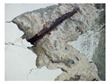 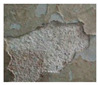 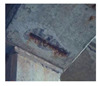 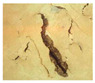
Brick masonry	Crack, rising dampness, biological growth, spalling, efflorescence, brick delamination, etc.	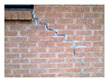 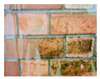 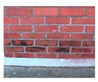 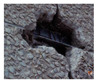
Plaster	Crack, biological growth, efflorescence, delamination, crazing, etc.	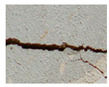 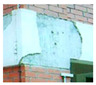 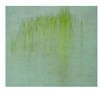 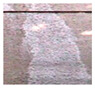
Tile	Crack, biological growth, efflorescence, chipping, tile buckling, tile delamination, staining, joint failure.	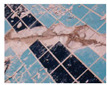 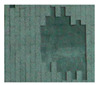 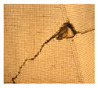 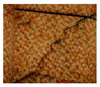
Stone cladding	Damaged/cracked cladding, inadequate support system, staining, uneven surface, etc.	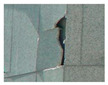 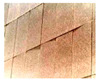 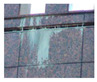 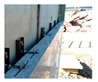
Metal cladding	Corrosion, inadequate support system, joint failure, biological staining, deformation buckling, etc.	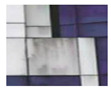 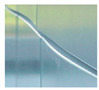 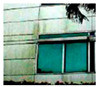 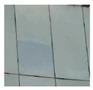
Glass cladding	Glass cracking, condensation, inadequate support system, joint failure, staining, etc.	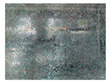 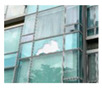 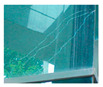 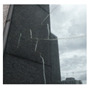

**Table 3:** Selected peer-reviewed articles on automated façade inspection.

Year	Reference	Brief Description of Work	Automation Devices	Data Acquisition Method
2010	[34]	Measure building façade dimensions with close-range photogrammetry	-	Image-based
2012	[35]	New flying voxel method for façade feature detection for generating a solid model to support computational modelling	-	Terrestrial laser scanning
2013	[22]	A low-cost aerial unit for outdoor geometric data acquisition and façade inspection	UAV	Image-based
2013	[36]	Combined 3D terrestrial laser scanning and total station surveying to detect façade damage	-	Terrestrial laser scanning
2013	[37]	Voxelisation and flying voxel method in reconstructing building models from LiDAR data	-	Terrestrial laser scanning
2015	[38]	Assessing pathologies in façades (Villamayor Stone) using a terrestrial laser scanner	-	Terrestrial laser scanning
2015	[39]	Use of multi-level image features and the feature matching method to characterise façades from typical urban scenes.	UAV	Image-based (aerial oblique images)
2015	[40]	Detection of delamination of adhered ceramic claddings using a thermography approach	-	Thermography
2015	[41]	Quasi-quantitative thermographic detection of moisture variation in façades with adhered ceramic cladding	-	Thermography
2016	[42]	Multi-spectral camera (530–801 nm) and terrestrial laser scanner (905 nm) for detecting different materials and damages on building façades	-	Image and LiDAR-based
2016	[43]	Analyse façade defects by studying the behaviour of Delta-T and contrast functions using infrared thermography	-	Thermography
2016	[44]	Qualitatively compares pass-by thermography and walk-through thermography for defect detection	-	Thermography
2016	[45]	Slicing method for curved façade and window extraction from point cloud data	-	Laser scanning
2017	[46]	Detection of damaged façade using local symmetry features and the Gini Index with aerial oblique images	UAV	Image-based (aerial oblique images)
2017	[47]	Assessing the capacity of thermography for detecting adhesion and analysing the influence of tile colour and support on inspection	-	Thermography
2018	[10]	Detecting concrete cracks in images acquired by unmanned aerial vehicles	UAV	Image-based
2019	[48]	Development of a façade-cleaning robot equipped with a deep-learning-based detection algorithm for crack identification	Cleaning Robot	Image-based
2019	[49]	Terrestrial laser scanning for detecting small damages on the brick façade	-	Terrestrial laser scanning
2020	[50]	New GIS-supported modelling method with multi-sourced image data for building façade inspections	UAV	Image-based
2020	[51]	Integrate multi-temporal aerial oblique image data with convolutional neural networks for façade damage detection	UAV	Image-based (aerial oblique images)
2020	[52]	Develop a region-based convolutional neural net to detect surface cracks, spalling and damage	-	Image-based
2020	[24]	Automatic layer classification method for floor plan and elevation detection to enable the reconstruction of a 3D (façade) BIM model	-	Image-based
2020	[26]	Meta-learning-based convolutional neural network for façade defects classification from the imbalanced dataset	-	Image-based
2020	[3]	Develop a deep-learning-based deblurring model to resolve motion blur due to the excessive vibrations of UAVs amid crack detection	UAV	Image-based
2020	[53]	A semi-supervised learning algorithm with a small amount of labelled data for façade defects classification	-	Image-based
2020	[54]	Supervised detection of façade windows and doors from photogrammetric 3D point clouds with RGB images and thermal infrared information	-	Thermal and RGB image
2021	[23]	Approach for geo-registering and managing UAV-collected images to the 2D GIS spatial model for façade inspection	UAV	Image-based
2021	[26]	A rule-based deep learning method to achieve evaluation-oriented façade defects detection	-	Image-based
2021	[55]	A two-step convolutional neural network method for the automated crack segmentation amid building façade inspections	UAV	Image-based
2021	[21]	Develop a thermal and RGB data-fusion framework to create a thermal mapping. Evaluate the impact of flight configurations on the data fusion (incl. façade detection)	UAV	Thermal and RGB image
2021	[56]	Assess decay phenomena and anomalies affecting the Cathedral façade through the evaluation of thermal and RGB images		Thermal and RGB image
2021	[57]	Present an automatic inspection method of building surfaces with the integration of UAVs and BIM	UAV	Image-based
2021	[58]	Present U-Net in pixelwise segmentation for defect detection including defect identification	-	Image-based
2021	[59]	A new automatic generation method for 3D building façade model reconstruction from the photogrammetric mesh	-	Image-based
2022	[60]	A bounding-box object augmentation method which enhances the automated defect detection in residential building façades	UAV	Image-based
2022	[61]	A hieratical deep learning framework to automatically detect building façade elements	-	Image-based
2022	[62]	Mask region-based convolutional neural networks for the automatic detection and segmentation of façade defects	-	Image-based
2022	[63]	Active infrared thermography for the segmentation of defect areas and automation in the thermal image processing	-	Thermography

**Table 4:** Advantages and disadvantages of various algorithms for DfM optimisation.

Optimisation Methods	Advantages	Disadvantages
Evolutionary optimisation algorithms	Optimisation for large numbers of variables Apply to both discrete and continuous variables Provide a sub-optimum which is more feasible for engineering problems	Results are sensitive to population size, crossover, mutation, etc. Computationally demanding for complex problems May have premature convergence
Particle swarm optimisation algorithms	As compared to evolutionary algorithms, fewer parameters are required Shorter computational time Higher efficiency for global searching	Converge prematurely leading to sub-optimum for complex problems Poor handing with a discrete variable optimisation
Harmony search	Easy for implementation	Require longer computation time due to the lack of global gradient
Ant colony algorithms	Rapid discovery of optimal solutions	Probability distribution changes iteratively Uncertainty for convergence
Neural network computing	Information stored through the network Learn from historical data and adapt to unknown situations	Results explainability due to the black box nature Difficulty of a good network structure

## Data Availability

Some or all of the data, models or code that support the findings of this study are available from the corresponding author upon reasonable request.

## References

[1] Guo J., Wang Q., Li Y., Liu P. (2020). Façade defects classification from imbalanced dataset using meta learning-based convolutional neural network. Comput.-Aided Civ. Infrastruct. Eng..

[2] Chew M.Y. (2021). Façade inspection for falling objects from tall buildings in Singapore. Int. J. Build. Pathol. Adapt..

[3] Liu Y., Yeoh J.K., Chua D.K. (2020). Deep learning–based enhancement of motion blurred UAV concrete crack images. J. Comput. Civ. Eng..

[4] Park H.S., Lee H., Adeli H., Lee I. (2007). A new approach for health monitoring of structures: Terrestrial laser scanning. Comput.-Aided Civ. Infrastruct. Eng..

[5] Guo J., Wang Q. (2022). Human-Related Uncertainty Analysis for Automation-Enabled Façade Visual Inspection: A Delphi Study. J. Manag. Eng..

[6] Ekanayake B., Wong J.K.-W., Fini A.A.F., Smith P. (2021). Computer vision-based interior construction progress monitoring: A literature review and future research directions. Autom. Constr..

[7] Kim M.-K., Sohn H., Chang C.-C. (2015). Localization and quantification of concrete spalling defects using terrestrial laser scanning. J. Comput. Civ. Eng..

[8] Mader D., Blaskow R., Westfeld P., Weller C. (2016). Potential of UAV-Based laser scanner and multispectral camera data in building inspection. Int. Arch. Photogramm. Remote Sens. Spat. Inf. Sci..

[9] Mukupa W., Roberts G.W., Hancock C.M., Al-Manasir K. (2017). A review of the use of terrestrial laser scanning application for change detection and deformation monitoring of structures. Surv. Rev..

[10] Zhong X., Peng X., Yan S., Shen M., Zhai Y. (2018). Assessment of the feasibility of detecting concrete cracks in images acquired by unmanned aerial vehicles. Autom. Constr..

[11] Tomita K., Chew M.Y.L. (2022). A Review of Infrared Th for Delamination Detection on Infrastructures and Buildings. Sensors.

[12] Chew M. (1992). The study of adhesion failure of wall tiles. Build. Environ..

[13] Shi Z., Ergan S. Towards point cloud and model-based urban façade inspection: Challenges in the urban façade inspection process. Proceedings of the Construction Research Congress 2020: Safety, Workforce, and Education.

[14] Zhou Z., Gong J., Guo M. (2016). Image-based 3D reconstruction for posthurricane residential building damage assessment. J. Comput. Civ. Eng..

[15] Chew M., Tan S., Kang K. (2005). Contribution analysis of maintainability factors for cladding facades. Archit. Sci. Rev..

[16] Chew M., De Silva N. (2004). Factorial method for performance assessment of building facades. J. Constr. Eng. Manag..

[17] Chew Y.L.M. (1998). Building Facades: A Guide to Common Defects in Tropical Climates.

[18] Chicago Department of Buildings (2016). Maintenance of High Rise Exterior Walls and Enclosures.

[19] Code of Ordinances Chapter 1127—General Inspection Programs. https://library.municode.com/oh/cincinnati/codes/code_of_ordinances?nodeId=TITXICIBUCO_CH1127GEINPR.

[20] Régie du Bâtiment du Québec Safety Code—Building Act. https://www.rbq.gouv.qc.ca/en/areas-of-intervention/building/technical-information/building-chapter-from-the-safety-code/facades-maintenance-and-inspection.html.

[21] Hou Y., Volk R., Chen M., Soibelman L. (2021). Fusing tie points’ RGB and thermal information for mapping large areas based on aerial images: A study of fusion performance under different flight configurations and experimental conditions. Autom. Constr..

[22] Roca D., Lagüela S., Díaz-Vilariño L., Armesto J., Arias P. (2013). Low-cost aerial unit for outdoor inspection of building façades. Autom. Constr..

[23] Chen K., Reichard G., Akanmu A., Xu X. (2021). Geo-registering UAV-captured close-range images to GIS-based spatial model for building façade inspections. Autom. Constr..

[24] Yin M., Tang L., Zhou T., Wen Y., Xu R., Deng W. (2020). Automatic layer classification method-based elevation recognition in architectural drawings for reconstruction of 3D BIM models. Autom. Constr..

[25] Chew Y.L.M. (2016). Maintainability of Facilities—Green FM for Building Professionals.

[26] Guo J., Wang Q., Li Y. (2021). Evaluation-oriented façade defects detection using rule-based deep learning method. Autom. Constr..

[27] (2019). Standard Practice for Periodic Inspection of Building Facades for Unsafe Conditions.

[28] (2019). Standard Guide for Conducting Inspections of Building Fcades for Unsafe Condition.

[29] Ohio Building & Housing Ordinances Exterior Wall and Appurtenances Inspections. https://www.clevelandohio.gov/CityofCleveland/Home/Government/CityAgencies/BuildingHousing/Ordinances.

[30] New York City Department of Buildings (1998). Local Law 11 of 1998.

[31] San Francisco Department of Buildings (2016). Building Code—Building Fa9ade In-Spection and Maintenance and Estab-Lishing Fee.

[32] Buildings Department (2017). Mandatory Building Inspection Scheme and Mandatory Window Inspection Scheme—Buildings (Amendment) Bill 2010.

[33] Singapore Statutes Online (1989). Building Control Act 1989.

[34] Ordóñez C., Martínez J., Arias P., Armesto J. (2010). Measuring building façades with a low-cost close-range photogrammetry system. Autom. Constr..

[35] Truong-Hong L., Laefer D.F., Hinks T., Carr H. (2012). Flying voxel method with Delaunay triangulation criterion for façade/feature detection for computation. J. Comput. Civ. Eng..

[36] Mill T., Alt A., Liias R. (2013). Combined 3D building surveying techniques–terrestrial laser scanning (TLS) and total station surveying for BIM data management purposes. J. Civ. Eng. Manag..

[37] Truong-Hong L., Laefer D.F., Hinks T., Carr H. (2013). Combining an angle criterion with voxelization and the flying voxel method in reconstructing building models from LiDAR data. Comput.-Aided Civ. Infrastruct. Eng..

[38] García Talegón J., Calabrés S., Fernández-Lozano J., Iñigo A.C., Herrero-Fernández H., Arias-Pérez B., González-Aguilera D. (2015). Assessing pathologies on villamayor stone (Salamanca, Spain) by terrestrial laser scanner intensity data. ISPRS.

[39] Yang X., Qin X., Wang J., Wang J., Ye X., Qin Q. (2015). Building Façade Recognition Using Oblique Aerial Images. Remote Sens..

[40] Edis E., Flores-Colen I., De Brito J. (2015). Building thermography: Detection of delamination of adhered ceramic claddings using the passive approach. J. Nondestruct. Eval..

[41] Edis E., Flores-Colen I., de Brito J. (2015). Quasi-quantitative infrared thermographic detection of moisture variation in facades with adhered ceramic cladding using principal component analysis. Build. Environ..

[42] Del Pozo S., Herrero-Pascual J., Felipe-García B., Hernández-López D., Rodríguez-Gonzálvez P., González-Aguilera D. (2016). Multispectral Radiometric Analysis of Façades to Detect Pathologies from Active and Passive Remote Sensing. Remote Sens..

[43] Bauer E., Pavon E., Barreira E., De Castro E.K. (2016). Analysis of building facade defects using infrared thermography: Laboratory studies. J. Build. Eng..

[44] Fox M., Goodhew S., De Wilde P. (2016). Building defect detection: External versus internal thermography. Build. Environ..

[45] Iman Zolanvari S.M., Laefer D.F. (2016). Slicing Method for curved façade and window extraction from point clouds. ISPRS J. Photogramm. Remote Sens..

[46] Tu J., Sui H., Feng W., Sun K., Xu C., Han Q. (2017). Detecting building facade damage from oblique aerial images using local symmetry feature and the GINI index. Remote Sens. Lett..

[47] Lourenço T., Matias L., Faria P. (2017). Anomalies detection in adhesive wall tiling systems by infrared thermography. Constr. Build. Mater..

[48] Kouzehgar M., Tamilselvam Y.K., Heredia M.V., Elara M.R. (2019). Self-reconfigurable façade-cleaning robot equipped with deep-learning-based crack detection based on convolutional neural networks. Autom. Constr..

[49] Masiero A., Costantino D. (2019). TLS for detecting small damages on a building façade. Int. Arch. Photogramm. Remote Sens. Spat. Inf. Sci..

[50] Chen K., Reichard G., Xu X. GIS-Based Modeling of Multi-Sourced Image Data Collected for Building Facade Inspection. Proceedings of the Construction Research Congress 2020: Computer Applications.

[51] Duarte D., Nex F., Kerle N., Vosselman G. (2020). Detection of seismic façade damages with multi-temporal oblique aerial imagery. GIScience Remote Sens..

[52] Ghosh Mondal T., Jahanshahi M.R., Wu R.T., Wu Z.Y. (2020). Deep learning-based multi-class damage detection for autonomous post-disaster reconnaissance. Struct. Control Health Monit..

[53] Guo J., Wang Q., Li Y. (2021). Semi-supervised learning based on convolutional neural network and uncertainty filter for façade defects classification. Comput.-Aided Civ. Infrastruct. Eng..

[54] Jarząbek-Rychard M., Lin D., Maas H.-G. (2020). Supervised Detection of Façade Openings in 3D Point Clouds with Thermal Attributes. Remote Sens..

[55] Chen K., Reichard G., Xu X., Akanmu A. (2021). Automated crack segmentation in close-range building façade inspection images using deep learning techniques. J. Build. Eng..

[56] Donato A., Randazzo L., Ricca M., Rovella N., Collina M., Ruggieri N., Dodaro F., Costanzo A., Alberghina M.F., Schiavone S. (2021). Decay Assessment of Stone-Built Cultural Heritage: The Case Study of the Cosenza Cathedral Façade (South Calabria, Italy). Remote Sens..

[57] Tan Y., Li S., Liu H., Chen P., Zhou Z. (2021). Automatic inspection data collection of building surface based on BIM and UAV. Autom. Constr..

[58] Jiang Y., Han S., Bai Y. (2021). Building and Infrastructure Defect Detection and Visualization Using Drone and Deep Learning Technologies. J. Perform. Constr. Facil..

[59] Zhang Y., Zhang C., Chen S., Chen X. (2021). Automatic Reconstruction of Building Façade Model from Photogrammetric Mesh Model. Remote Sens..

[60] Lee K., Lee S., Kim H.Y. (2022). Bounding-box object augmentation with random transformations for automated defect detection in residential building façades. Autom. Constr..

[61] Zhang G., Pan Y., Zhang L. (2022). Deep learning for detecting building façade elements from images considering prior knowledge. Autom. Constr..

[62] Li J., Wang Q., Ma J., Guo J. (2022). Multi-defect segmentation from façade images using balanced copy–paste method. Comput.-Aided Civ. Infrastruct. Eng..

[63] Garrido I., Barreira E., Almeida R.M., Lagüela S. (2022). Introduction of active thermography and automatic defect segmentation in the thermographic inspection of specimens of ceramic tiling for building façades. Infrared Phys. Technol..

[64] Eastman C.M., Eastman C., Teicholz P., Sacks R., Liston K. (2011). BIM Handbook: A Guide to Building Information Modeling for Owners, Managers, Designers, Engineers and Contractors.

[65] Kim M.-K., Wang Q., Li H. (2019). Non-contact sensing based geometric quality assessment of buildings and civil structures: A review. Autom. Constr..

[66] Paneru S., Jeelani I. (2021). Computer vision applications in construction: Current state, opportunities & challenges. Autom. Constr..

[67] Han K., Degol J., Golparvar-Fard M. (2018). Geometry-and appearance-based reasoning of construction progress monitoring. J. Constr. Eng. Manag..

[68] Yu S.-N., Jang J.-H., Han C.-S. (2007). Auto inspection system using a mobile robot for detecting concrete cracks in a tunnel. Autom. Constr..

[69] Menendez E., Victores J.G., Montero R., Martínez S., Balaguer C. (2018). Tunnel structural inspection and assessment using an autonomous robotic system. Autom. Constr..

[70] Koch C., Georgieva K., Kasireddy V., Akinci B., Fieguth P. (2015). A review on computer vision based defect detection and condition assessment of concrete and asphalt civil infrastructure. Adv. Eng. Inform..

[71] Agnisarman S., Lopes S., Chalil Madathil K., Piratla K., Gramopadhye A. (2019). A survey of automation-enabled human-in-the-loop systems for infrastructure visual inspection. Autom. Constr..

[72] Dias I.S., Flores-Colen I., Silva A. (2021). Critical Analysis about Emerging Technologies for Building’s Façade Inspection. Buildings.

[73] Bolourian N., Hammad A. (2020). LiDAR-equipped UAV path planning considering potential locations of defects for bridge inspection. Autom. Constr..

[74] Lins R.G., Givigi S.N., Freitas A.D., Beaulieu A. (2016). Autonomous robot system for inspection of defects in civil infrastructures. IEEE Syst. J..

[75] Asadi K., Kalkunte Suresh A., Ender A., Gotad S., Maniyar S., Anand S., Noghabaei M., Han K., Lobaton E., Wu T. (2020). An integrated UGV-UAV system for construction site data collection. Autom. Constr..

[76] Chi H.-L., Wang X., Jiao Y. (2015). BIM-Enabled Structural Design: Impacts and Future Developments in Structural Modelling, Analysis and Optimisation Processes. Arch. Comput. Methods Eng..

[77] Son H., Bosché F., Kim C. (2015). As-built data acquisition and its use in production monitoring and automated layout of civil infrastructure: A survey. Adv. Eng. Inform..

[78] Pătrăucean V., Armeni I., Nahangi M., Yeung J., Brilakis I., Haas C. (2015). State of research in automatic as-built modelling. Adv. Eng. Inform..

[79] Yin C., Cheng J.C.P., Wang B., Gan V.J.L. (2022). Automated classification of piping components from 3D LiDAR point clouds using SE-PseudoGrid. Autom. Constr..

[80] Brilakis I., Lourakis M., Sacks R., Savarese S., Christodoulou S., Teizer J., Makhmalbaf A. (2010). Toward automated generation of parametric BIMs based on hybrid video and laser scanning data. Adv. Eng. Inform..

[81] Tang P., Huber D., Akinci B., Lipman R., Lytle A. (2010). Automatic reconstruction of as-built building information models from laser-scanned point clouds: A review of related techniques. Autom. Constr..

[82] Xiong X., Adan A., Akinci B., Huber D. (2013). Automatic creation of semantically rich 3D building models from laser scanner data. Autom. Constr..

[83] Santos R., Costa A.A., Grilo A. (2017). Bibliometric analysis and review of Building Information Modelling literature published between 2005 and 2015. Autom. Constr..

[84] Sacks R., Kaner I., Eastman C.M., Jeong Y.-S. (2010). The Rosewood experiment—Building information modeling and interoperability for architectural precast facades. Autom. Constr..

[85] Tan Y., Li G., Cai R., Ma J., Wang M. (2022). Mapping and modelling defect data from UAV captured images to BIM for building external wall inspection. Autom. Constr..

[86] Liu Y., Li M., Wong B.C.L., Chan C.M., Cheng J.C.P., Gan V.J.L. (2021). BIM-BVBS integration with openBIM standards for automatic prefabrication of steel reinforcement. Autom. Constr..

[87] Sacks R., Ma L., Yosef R., Borrmann A., Daum S., Kattel U. (2017). Semantic enrichment for building information modeling: Procedure for compiling inference rules and operators for complex geometry. J. Comput. Civ. Eng..

[88] Motamedi A., Yabuki N., Fukuda T. Extending BIM to include defects and degradations of buildings and infrastructure facilities. Proceedings of the 3rd International Conference on Civil and Building Engineering Informatics in conjunction with 2017 Conference on Computer Applications in Civil and Hydraulic Engineering (ICCBEI & CCACHE 2017).

[89] Artus M., Alabassy M.S.H., Koch C. (2022). A BIM Based Framework for Damage Segmentation, Modeling, and Visualization Using IFC. Appl. Sci..

[90] Hao Q., Xue Y., Shen W., Jones B., Zhu J. A decision support system for integrating corrective maintenance, preventive maintenance, and condition-based maintenance. Proceedings of the Construction Research Congress 2010: Innovation for Reshaping Construction Practice.

[91] Zhang F., Chan A.P.C., Darko A., Chen Z., Li D. (2022). Integrated applications of building information modeling and artificial intelligence techniques in the AEC/FM industry. Autom. Constr..

[92] Fang Q., Li H., Luo X., Ding L., Luo H., Li C. (2018). Computer vision aided inspection on falling prevention measures for steeplejacks in an aerial environment. Autom. Constr..

[93] Wu W., Yang H., Li Q., Chew D. (2013). An integrated information management model for proactive prevention of struck-by-falling-object accidents on construction sites. Autom. Constr..

[94] Klimkowska A., Cavazzi S., Leach R., Grebby S. (2022). Detailed Three-Dimensional Building Façade Reconstruction: A Review on Applications, Data and Technologies. Remote Sens..

[95] Alavi H., Bortolini R., Forcada N. (2022). BIM-based decision support for building condition assessment. Autom. Constr..

[96] Chen W., Chen K., Cheng J.C.P., Wang Q., Gan V.J.L. (2018). BIM-based framework for automatic scheduling of facility maintenance work orders. Autom. Constr..

[97] Vieira S.M., Silva A., Sousa J.M.C., de Brito J., Gaspar P.L. (2015). Modelling the service life of rendered facades using fuzzy systems. Autom. Constr..

[98] Flores-Colen I., de Brito J. (2010). A systematic approach for maintenance budgeting of buildings façades based on predictive and preventive strategies. Constr. Build. Mater..

[99] Hallaji S.M., Fang Y., Winfrey B.K. (2022). Predictive maintenance of pumps in civil infrastructure: State-of-the-art, challenges and future directions. Autom. Constr..

[100] Errandonea I., Beltrán S., Arrizabalaga S. (2020). Digital Twin for maintenance: A literature review. Comput. Ind..

[101] Gan V.J.L. (2022). BIM-based graph data model for automatic generative design of modular buildings. Autom. Constr..

[102] Gan V.J.L., Wong C.L., Tse K.T., Cheng J.C.P., Lo I.M.C., Chan C.M. (2019). Parametric modelling and evolutionary optimization for cost-optimal and low-carbon design of high-rise reinforced concrete buildings. Adv. Eng. Inform..

[103] Boonstra S., van der Blom K., Hofmeyer H., Emmerich M.T.M. (2021). Hybridization of an evolutionary algorithm and simulations of co-evolutionary design processes for early-stage building spatial design optimization. Autom. Constr..

[104] Gan V.J.L., Wang B., Chan C.M., Weerasuriya A.U., Cheng J.C.P. (2021). Physics-based, data-driven approach for predicting natural ventilation of residential high-rise buildings. Build. Simul..

[105] Weerasuriya A.U., Zhang X., Gan V.J.L., Tan Y. (2019). A holistic framework to utilize natural ventilation to optimize energy performance of residential high-rise buildings. Build. Environ..

[106] Liao W., Lu X., Huang Y., Zheng Z., Lin Y. (2021). Automated structural design of shear wall residential buildings using generative adversarial networks. Autom. Constr..

[107] Ghannad P., Lee Y.-C. (2022). Automated modular housing design using a module configuration algorithm and a coupled generative adversarial network (CoGAN). Autom. Constr..

[108] Heidari Matin N., Eydgahi A. (2021). A data-driven optimized daylight pattern for responsive facades design. Intell. Build. Int..

[109] Moghtadernejad S., Chouinard L.E., Mirza M.S. (2021). Enhanced façade design: A data-driven approach for decision analysis based on past experiences. Dev. Built Environ..

[110] Montali J., Sauchelli M., Jin Q., Overend M. (2019). Knowledge-rich optimisation of prefabricated façades to support conceptual design. Autom. Constr..

[111] Building and Construction Authority (BCA) (2019). Design for Maintainability Guide: Non-Residential.

[112] Balling R.J., Yao X. (1997). Optimization of reinforced concrete frames. J. Struct. Eng..

[113] Colin M., MacRae A. (1984). Optimization of structural concrete beams. J. Struct. Eng..

[114] Kanagasundaram S., Karihaloo B. (1990). Minimum cost design of reinforced concrete structures. Struct. Optim..

[115] Kanagasundaram S., Karihaloo B. (1991). Minimum-cost design of reinforced concrete structures. Comput. Struct..

[116] Rajeev S., Krishnamoorthy C.S. (1998). Genetic algorithm–based methodology for design optimization of reinforced concrete frames. Comput.-Aided Civ. Infrastruct. Eng..

[117] Esfandiari M.J., Urgessa G.S., Sheikholarefin S., Manshadi S.H.D. (2018). Optimum design of 3D reinforced concrete frames using DMPSO algorithm. Adv. Eng. Softw..

[118] Esfandiary M.J., Sheikholarefin S., Rahimi Bondarabadi H.A. (2016). A combination of particle swarm optimization and multi-criterion decision-making for optimum design of reinforced concrete frames. Int. J. Optim. Civ. Eng..

[119] Akin A., Saka M.P. (2015). Harmony search algorithm based optimum detailed design of reinforced concrete plane frames subject to ACI 318-05 provisions. Comput. Struct..

[120] Kaveh A., Talatahari S. (2010). An improved ant colony optimization for the design of planar steel frames. Eng. Struct..

[121] Mangal M., Li M., Gan V.J.L., Cheng J.C.P. (2021). Automated clash-free optimization of steel reinforcement in RC frame structures using building information modeling and two-stage genetic algorithm. Autom. Constr..

[122] Masouleh K.B. (2018). Building Energy Optimisation Using Machine Learning and Metaheuristic Algorithms. Ph.D. Thesis.

[123] Gan V.J. (2022). BIM-Based Building Geometric Modeling and Automatic Generative Design for Sustainable Offsite Construction. J. Constr. Eng. Manag..

[124] As I., Pal S., Basu P. (2018). Artificial intelligence in architecture: Generating conceptual design via deep learning. Int. J. Archit. Comput..

[125] Nauata N., Chang K.-H., Cheng C.-Y., Mori G., Furukawa Y. House-gan: Relational generative adversarial networks for graph-constrained house layout generation. Proceedings of the European Conference on Computer Vision.

[126] Newton D. (2019). Generative deep learning in architectural design. Technol. Archit. Des..

